# Exploring the needs of family caregivers of people living with mental illness: a qualitative study in Lobatse, Botswana

**DOI:** 10.1186/s12912-025-02813-7

**Published:** 2025-02-17

**Authors:** Keletwaetse Sakwape, Gaotswake Patience Kovane, Precious Chibuike Chukwuere, Miriam Mmamphamo Moagi, Rorisang Machailo

**Affiliations:** 1 NuMIQ Research Focus Area, Faculty of Health Sciences, North-West University, Potchefstroom, South Africa; 2 Faculty of Health Sciences, School of Nursing, University of Botswana, Gaborone, Botswana; 3 Nursing Department, University of Limpopo, Sovenga, Limpopo Province South Africa

**Keywords:** Explore, Family caregivers, Mental illness, Needs, Support

## Abstract

**Background:**

Mental disorders significantly contribute to the global disease burden and are a leading cause of disability worldwide. 57.8 million adults deal with some form of mental condition. Family caregivers play a crucial role in supporting people living with mental illness (PLWMI), but their own well-being is often impacted. When a family member has a mental illness, caregivers often feel solely responsible for various aspects, significantly increasing the burden of caregiving. This study aimed to explore and describe the needs of family caregivers of people living with mental illness in Lobatse.

**Methods:**

The study used an exploratory, descriptive, contextual qualitative design to explore and describe the needs of family members caring for relatives diagnosed with mental illnesses. A purposive sampling technique was used to select the family caregivers who participated in the study. Data was collected through in-depth individual face-to-face interviews and analysed using a content analysis approach. The study’s trustworthiness was ensured by establishing credibility, transferability, authenticity, confirmability, and dependability. Approval to conduct the study was obtained from the relevant authorities, and written informed consent was secured from all participants.

**Results:**

Several key needs of PLWMI family caregivers were identified: the need for interventions to assist family caregivers, the need for informational support, and the need for perceptible support.

**Conclusion:**

Caring for a family member with mental illness at home is highly demanding and has negative effects on the caregivers’ financial, social, and emotional well-being. The study uncovered the specific needs of family caregivers. These findings underscore the importance of developing guidelines to improve the well-being of family caregivers and provide them with necessary support.

**Supplementary Information:**

The online version contains supplementary material available at 10.1186/s12912-025-02813-7.

## Introduction

Mental disorders significantly contribute to the global disease burden and stand as a leading cause of disability worldwide, accounting for a substantial proportion of years lived with disability [1]. 57.8 million adults are suffering from some form of mental condition [2]. Mental illness affects not only the patient but also the psychosocial functioning of their family and friends [3]. While the deinstitutionalization of psychiatric patients is crucial for honouring their autonomy, statistics from developing countries reveal that many patients are discharged even when they still require care, largely due to the scarcity of available psychiatric facilities [4, 5]. Thus, they regularly receive support and care in home settings from their family members [6]. Therefore, family caregivers are highly regarded as an essential resource in the support and care of people living with mental illness (PLWMI) [7]. Involving caregivers in treating and caring for PLWMI can positively impact patient health [8, 9]. However, the caregivers bear a negative caregiving burden [10].

Research indicates a strong emphasis on family caregivers’ physical and mental well-being. However, it’s crucial to recognize that caregiving also significantly impacts the health of the individual receiving care [5]. Evolving research indicates that in most cases, family caregivers focus on the well-being of their ill relatives at the expense of their own needs [11]. Studies suggest that the provision of care to PLWMI has an impact not only on the psychological and physical health of the family caregivers but also their ability to cope with the caregiving strain, conferring the risk of mental and physical illness such as depression and hypertension, respectively [12, 13].

The presence of a person diagnosed with mental illness in the family leads most family caregivers to have a feeling of taking the sole responsibility for various financial, personal, and home life aspects, thereby significantly contributing to the rise in caregiving burden [11]. Moreover, the family caregivers provide the patient with emotional and physical support and endure the physical and emotional strain emanating from the patient’s disruptive behaviours that consequently impact the daily routines and aptitude to carry out usual social activities [14]. The caregivers relentlessly supervise their mentally ill loved ones, having difficulty balancing the caregiving demands and handling their daily life, which encompasses carrying out many responsibilities simultaneously [11]. As a result, there is increasing worry about their aptitude to manage and cope due to the cumulative responsibilities and demands [15].

The burden incurred by the family caregivers of PLWMI can be subjective or objective. Subjective burden refers to psychological strain such as rejection, sadness, guilt, loss, and anger [16]. Meanwhile, objective burden involves caregivers’ employment and financial challenges, social isolation, and disruption of the caregivers’ daily activities. The family caregiver burden is anticipated to be aggravated in settings deficient in other community-based supports [17].

Literature underscores the extent of the global family caregiver burden. In a study conducted in India, the severe burden due to family caregiving accounted for 40.9%, and the moderate burden was 59.1% among family caregivers [15]. Similarly, in Egypt, caregivers experienced a high burden, with the mean score of the Zarit burden scale as 55.20 ± 9.82 [18]. Meanwhile, in sub-Saharan Africa, studies indicate comparably high levels of caregiving burden. The burden of caring for PLWMI in various countries of the sub-Saharan region ranges from 60 to 90% [6]. In Nigeria, the prevalence of the burden of family caregiving was 85.3%, with 51.3% being mild-to-moderate and 34.0% being high/or severe burden [5]. Furthermore, Ethiopia’s global objective burden score indicates an approximate 72.9% moderate to severe burden experienced by family caregivers of PLWMI [6]. In a study conducted in rural Kwa-Zulu Natal in South Africa, most family caregivers experienced mild/moderate levels of burden (39.50%), 35.01% reported moderate/severe levels, and 15.13% experienced severe levels of caregiver burden [19].

Since the 1970s, the approach to caring for PLWMI in Botswana has evolved significantly [20]. The treatment of individuals diagnosed with mental illness has undergone significant evolution over time, advancing through various phases. These phases include the medieval pre-penal era, the penal era, and the institutionalization period. Today, the focus has shifted to a model of deinstitutionalized, community-based care that emphasizes collaboration with clients and their active involvement in the treatment process [21, 22]. One of the most evident outcomes of this deinstitutionalization in Botswana is the increase in challenges related to community-based psychiatric care. As a result, many families with relatives who have mental illness often find themselves assuming the caregiving role [20].

Increasing recognition of the needs of family caregivers has prompted service providers and governments in several countries to invest in programmes and support interventions to assist caregivers to handle the strain associated with their caregiving role [23, 24]. A study by Gharavi et al. [25] indicates that families exposed to the Interaction-Skills Training programme had a significantly decreased burden and experienced a greater sense of competence in caring for their patients with severe mental illness. Furthermore, Navidian and colleagues suggested that psychoeducation interventions reduced the burden on family caregivers [26]. Additionally, in a study conducted by Desouki et al. [27], a programme aimed at reducing the burden of caregivers of patients diagnosed with schizophrenia yielded positive results i.e. reduction in burden and incompetence. Thus, it can be concluded through empirical evidence that interventions like programmes and guidelines reduce the burden of caregiving [27, 28]. Although there is growing literature on the effect of mental illness on families globally, there is still a paucity of published data relating to the family caregiver burden and their needs in low-income countries such as Botswana. Family caregivers provide their mentally ill relatives with various support services, including physical, financial, and emotional support.

In Botswana, caregivers often endure feelings of guilt and courtesy stigma associated with mental illness from the public. This social stigma frequently leads to a lack of supportive social networks for these caregivers [13, 29]. The Ministry of Health (MoH) in Botswana regularly conducts workshops and seminars to educate both caregivers and healthcare professionals on how to effectively support caregivers. However, some critical aspects of caregiver needs are not sufficiently addressed. The existing interventions do not provide context-specific solutions. As a result, the researcher aimed to explore the needs of family caregivers with the aim to develop tailored support guidelines and programs for caregivers in Lobatse.

## Methods

### Study design

An exploratory, descriptive, contextual qualitative study design was employed to gather deeper insight into the needs of family members caring for their relatives diagnosed with mental illnesses. This qualitative research design was envisioned to examine and describe an occurrence that needs clarity as it naturally occurs in its original setting [30]. The design aided in discovering an in-depth understanding of the needs of family caregivers in their caregiving endeavour.

### Study setting

The study was conducted in Lobatse. Lobatse is a town located in the Southeast district of Botswana. The town is surrounded by mountains, in a valley moving in the northern direction towards Gaborone and near the border with South Africa. Lobatse is an administrative area with a town council, low to high-cost residential accommodations, and small to large-scale agricultural farms in the neighbouring areas. It is a key transit point for momentary trade travelling from South Africa, with two nearby border posts, Pioneer Border (9 km drive from Lobatse) on the way to Zeerust and Ramatlabama (54.3 km drive from Lobatse) border on the way to Mahikeng. The town is home to five clinics that offer outpatient services for individuals diagnosed with mental illnesses where the study was conducted. Lobatse has gained a strong reputation for its mental health services, owing to its long-standing history of providing specialized psychiatric care. This legacy began with Dr Sbrana, the country’s first psychiatrist, who established these services [31].

### Population and sampling

This study’s participants comprised 15 family caregivers of PLWMI. The family caregivers were purposively selected from five clinics in the Lobatse district health management team (LDHMT).

The study’s inclusion criteria comprised any family member, friend, or guardian staying with and caring for a person with a mental illness. The caregiver should have been 18 years or older and cared for the patient for over six months. Only one caregiver in a family participated in the study, and the caregivers should have been able to communicate in either English or Setswana.

The selection of participants excluded all caregivers temporarily taking care of the PLWMI, e.g., those employed to take care of the patients part-time, either during the day or at night.

### Recruitment

Recruitment was conducted by nurse managers, who were chosen as mediators by the LDHMT coordinator, the gatekeeper. The nurse managers recruited the family caregivers during morning meetings and consultations. The individuals who were purposively selected to participate in the study could communicate in either Setswana or English, as both are official languages and a key medium of communication in Botswana. All clients speak Setswana and another language.

The family caregivers who were purposively recruited and displayed interest in participating in the study were then directed to the independent person for consent negotiation. The independent person provided them further with detailed information about the study before signing the consent. The participants’ rights were explained to them, and they were told that participation was voluntary and that they had the right to withdraw anytime they wished without prejudice. They were given 24 hours to decide on participation and signed the consent form. The independent person received the participants’ contact details for follow-up for consent signing and an appointment for an interview if they were interested in participating in the study. The family caregivers who participated in the study also consented to be audio recorded and for filed notes to be taken.

### Data collection

In-depth, face-to-face interviews were conducted with family caregivers of PLWMI to gather comprehensive information. Each interview lasted 45 minutes to 1 hour and was held in the caregiver’s preferred language in a private room at the clinic, scheduled at a convenient time. Data were collected using a semi-structured interview guide with open-ended questions supplemented by reflective follow-ups to explore caregiver needs. The interview guide was developed inline with the aim of the study and pilot tested by the authors. The interviews were audio-recorded with participants’ permission, and reflective notes were taken. Fifteen caregivers participated in the study, and data saturation was reached after the 14th interview. A pilot interview providing valuable insights was also included in the analysis. At the end of each session, caregivers could add or clarify details.

### Data analysis

The tape-recorded interviews were then transcribed verbatim. Data analysis commenced from the first interview, as supported by literature regarding data analysis in qualitative studies [32]. Data analysis was conducted using the content analysis process comprising data extraction, organization, and synthesis. The four steps of the content analysis method, which entails “decontextualisation, recontextualisation, categorisation and compilation”, were utilized [33]. In addition, an independent co-coder was provided with the protocol for the steps to be followed for analysing the data to ensure the credibility of the results. The first author and the co-coder reached a consensus on the themes and sub-themes of the data, after which the transcriptions and the analysed data were submitted to the other three authors for verification. The co-coder signed a confidentiality agreement, affirming her commitment to adhering to the ethical standards.

### Ethical considerations

The authors obtained approval to conduct this study from the North-West University Health Research Ethics Committee (NWU-HREC), NWU-00178-23-A1. This university approval was then used to secure permission from the MoH, reference number HPRD: 6/14/1 and the Local District Health Management Team (LDHMT), reference GLDHMT 6/17/II, in Botswana to proceed with the study. To safeguard participants’ confidentiality, the authors informed them that the information collected would be used exclusively for this study and would not be shared with others without their consent. Privacy measures were further enhanced by conducting the interviews in a private office. Identifying information was removed from the data throughout the study to protect participants’ privacy and confidentiality. In addition, documents containing participants’ information were encrypted and required a password for access. Participants were also informed that the data collected would be sent to the university for storage in accordance with university policies.

### Data management

The audio recordings from the interviews were transferred to a password-protected laptop immediately after each interview was concluded, and thereafter, the recordings were deleted from the audio recorder. After transcribing the data, the audio files were stored in NextCloud, an electronic data storage system supported by the university’s information technology department. All electronic data received from participants were stored on a password-protected laptop. Hard copies of the data were kept in lockable cupboards within a secured room, under the supervision of the research coordinator, and would be shredded in accordance with university policy. According to this policy, the data must be retained at the university for five years before it can be safely destroyed.

### Trustworthiness

This study ensured trustworthiness by attentively examining the truthfulness of data and honouring participants’ meanings. Lincoln and Guba’s epistemological standards were applied to enhance rigour [30]. Credibility was strengthened through prolonged engagement with family caregivers, which helped us build trust and gain insights into their needs [34]. Confirmability was maintained by keeping a reflective journal throughout the research process and ensured dependability through an audit trail documenting all activities [35]. A comprehensive description of the research setting and methods supported transferability, allowing readers to assess applicability to similar contexts [34]. To ensure authenticity, all interviews followed a consistent guide and included diverse participants, with an audit trail of the audio-recorded interviews maintained [36].

## Results

### Demographic characteristics

A total of fifteen family caregivers of individuals living with mental illness (PLWMI) participated in the study. Among these participants, two were males, while thirteen were females. All participants identified as Black, aged 44 to 80 years and caregiving durations from 4 to 28 years. Among these participants, two had never attended school, one received non-formal education, three completed primary education, five finished secondary education, and four obtained tertiary education. The most common diagnosis among the PLWMI being cared for was substance use disorders (*n* = 6), followed by schizophrenia (*n* = 3), dementia (*n* = 2), and one case each of depression, attention deficit hyperactivity disorder, schizoaffective disorder, and bipolar mood disorder. Among the caregivers, nine were mothers, three were daughters, and the remaining caregivers included a partner, an aunt, and a father, as indicated in Table 1.

**Table 1:** Participants’ demographic profile

Participant	Age	Gender	Level of education	Relationship to patient	Duration of care	Diagnosis of the patient
1	54	Female	Secondary	Daughter	7 years	Dementia
2	58	Female	Primary	Mother	5 years	Substance use disorder
3	80	Male	None	Father	25 years	Schizophrenia
4	47	Male	Tertiary	Fiancé	4 years	Bipolar mood disorder
5	44	Female	Secondary	Mother	5 years	Substance use disorder
6	70	Female	None	Mother	24 years	Schizophrenia
7	48	Female	Secondary	Daughter	28 years	Attention deficit hyperactivity disorder
8	74	Female	Primary	Mother	15 years	Schizoaffective disorder
9	59	Female	Tertiary	Mother	16 years	Schizophrenia
10	75	Female	Primary	Mother	25 years	Substance use disorder, Schizophrenia
11	53	Female	Tertiary	Aunt	10 years	Substance use disorder
12	60	Female	Secondary	Mother	5 years	Depression
13	65	Female	Tertiary	Daughter	7 years	Dementia
14	73	Female	Non-formal education	Mother	10 years	Substance use disorder
15	57	Female	Secondary	Mother	5 years	Substance use disorder

### Organisation of themes and categories

The family caregivers’ responses on their needs in caring for PLWMI yielded the following themes and subthemes as presented in Table 2 below.

**Table 2:** Themes and categories

Themes	Categories
1. The need for interventions to assist family caregivers.	1.1. Assessment of family needs and resources 1.2. Home visits to assess and support family members. 1.3. Counselling the family caregivers to deal with the psychological strain.
2. Family caregivers need for informational support.	2.1. Informational needs and gaps 2.2. Skills training on the management of mental illness
3. Family caregivers’ need for perceptible support.	3.1. Grants and allowances for families living with mental illness 3.2. Support to access mental healthcare 3.3. Respite care to relieve family members’ burden

### Theme 1: the need for interventions to assist family caregivers

During interviews, the family caregivers reported needing interventions geared to help them cope with the strain of caring for PLWMI. They were worried that mental health professionals’ attention is focused only on patients, and no one cares about them. The sub-themes and quotes supporting the theme are discussed below.

#### Category 1.1: the need to assess the family caregivers

The family caregivers expressed the need to be assessed to evaluate their well-being and determine how they can be supported in caring for PLWMI. They indicated that caregiving of PLWMI takes a toll on their quality of life and that they would appreciate the interventions by mental health personnel, which should begin with assessing them. Their assertions are supported by the quotes below:

*“We should be assessed… it could have been a good thing because we are going through a lot*,* especially us who are caregivers of people living with mental illness.”* (P2, F, Clinic 5).

*“I really need this assessment. I feel I need to be assessed in the mind if there is any test of that sought. Taking care of a person diagnosed with mental illness is painful. I have a feeling it has affected me mentally. In short*,* we need to be assessed. Our hearts need to be checked. We are not leading a normal life.”* (P4, M, Clinic 3).

*“We need scheduled medical examinations specific to the caregivers. The job we are doing is heavy. We don’t have time for ourselves. We get assessed when not well*,* not relating to our caregiving role.”* (P7, F, Clinic 1).

#### Category 1.2: the need to conduct home visits to assess and support family members

The family caregivers expressed the need to be checked at home so that health professionals can conduct a holistic assessment of the family caregiver and their environment. The quotes below support the sub-theme.

*“The main issue is for health workers to educate the family members*,* during home visits that if there is a patient in the family*,* be it a parent or sibling*,* it should be a responsibility for all family members. It should not be the sole responsibility of the one who is not working.”* (P1, F, Clinic 2).

*“It would relieve my stress. When the time for his monthly reviews approaches*,* I get so stressed thinking about how I would do it. If he can be checked from home*,* it would relieve me. It can also assist in assessing the situation at home*,* and determining what can be done to support both of us. Maybe it can make health professionals advocate for us for the grant after seeing the situation at home.”* (P5, F, Clinic 4).

*“But they can be helpful to us. If nurses could check us in our homes*,* we could be able to tell them how we feel. In addition*,* they can see the environment we live in with our patients. Seeing that they care about our wellbeing could soothe us emotionally.”* (P6, F, Clinic 1).

#### Category 1.3: the need to counsel the family caregivers to deal with the psychological strain

Most family caregivers conveyed the need for counselling to relieve them of the emotional burden resulting from caring for their relatives diagnosed with mental illness.

*“We need to be counselled at least once or twice a month. Every day I meet different challenges in caring for her. Even if one tries to suppress their emotions*,* it is hard. So*,* counselling can help us overcome the stressful feelings we experience.”* (P4, M, Clinic 3).

*“Now*,* I need counselling from professionals*,* who can help me with tackling certain situations I am facing.”* (P9, F, Clinic 2).

*“I haven’t gotten any counselling*,* but I need emotional support. I want someone who can continuously talk to me. I don’t mind checking them to counsel me if they are there at the clinic. I just need someone who can give me time to express my emotions. My chest is full.”* (P11, F, Clinic 5).

### Theme 2: family caregivers need for informational support

The family caregivers expressed the need to understand the mental illness their relatives are diagnosed with. They reported taking care of patients whom they do not have insight into the condition they are suffering from.

#### Category 2.1: the need for psychoeducation and close informational gaps

The family caregivers reported that they do not have a clear understanding of the mental conditions their patients are diagnosed with. They wished they could get enough information to get more insight into the mental illnesses their relatives are diagnosed. Their assertions are supported by the quotes below:

*“I haven’t received any education about it. My son was admitted to a psychiatric hospital. He was discharged only recently*,* but I haven’t gotten any education. They only informed me about when to give him his medication. That’s the only thing they taught me. They didn’t tell me about the desired and side effects.”* (P10, F, Clinic 5).

*“After the diagnosis*,* she was admitted to the hospital. However*,* I was never provided with any education regarding her mental illness. She was given medication*,* which she takes every month. I just make sure she doesn’t miss her medication.”* (P12, F, clinic 2).

*“I have realized that we start this caregiving role without anyone telling us what to expect. I begin with the knowledge that they have been diagnosed with such an illness*,* not knowing exactly what it holds for me”* (P13, F, Clinic 4).

#### Category 2.2. The need to be capacitated on how to manage their family members diagnosed with mental illness at home

The family caregivers expressed their desire to be taught how to manage their relatives diagnosed with mental illness at home. They reported experiencing confusion when the patient changes behaviour at home.

*“I need to be taught what to expect from my son due to his illness. I need to understand how I should treat him at home and how to live with him without aggravating his illness.”* (P11, F, Clinic 5).

*“I would like to be taught to understand and accept people diagnosed with mental illness. I need to understand how to manage my patient*,* what he needs*,* and what he doesn’t want.”* (P14, F, Clinic 4).

### Theme 3: family caregivers’ need for perceptible support

The family caregivers reported the need for tangible support that can relieve them of the stress of caring for an individual diagnosed with mental illness.

#### Category 3.1: the need for financial support for families living with mental illness

Family members caring for their relatives diagnosed with mental illness expressed their desire to be supported financially to meet the demands of their caregiving role. The quotes below support them.

*“I need financial assistance to cover the various expenses I have. There are water and electricity bills*,* food and toiletries for the family*,* clothing and other necessities. I need many things I used to cover easily while working.”* (P3, M, Clinic 2).

*“They should give us monthly money to help reduce our financial burden. I don’t know what else I can say.”* (P6, F, Clinic 1).

*“The government should assist us with money to do this and that*,* especially to buy food for these children. They eat a lot*,* and it strains me to feed them*,* the money I have is not enough to feed the whole family”* (P8, F, Clinic 3).

#### Category 3.2: the need to be supported to access mental healthcare

The participants reported facing a challenge in accessing mental health services due to various factors, such as the distance between their homes and their facilities. Hence, they need support to access mental health services. Below are what the caregivers said regarding the above-mentioned need.

*“I’m struggling with his transportation. I use his old-age pension money to buy the diapers he uses and for all other necessities.”* (P15, F, Clinic 1).

*“It would reduce my stress concerning my daughter’s transportation to the clinic. When her review date approaches*,* I get so drained that I wonder how to take her to the clinic. This transport issue is a thorn in my heart.”* (P7, F, Clinic 1).

*“Now*,* money for transport or taxis is a challenge because I’m not working. I’m just staying at home. I’m faced with the challenge of giving him money for transport to the clinic for review and to collect his medication. When I don’t have money*,* it becomes difficult for him to go to the clinic.”* (P9, F, Clinic 2).

#### Category 3.3. The need for respite care centres to relieve family members’ burden

The family caregivers voiced out their wish to get relief from caregiving. They wished there was somewhere that could constantly be used to accommodate their patients temporarily as they take a breather in the caregiving role. The quotes below support their expressions:

*“The government or town council should build centres where we can drop them off temporarily while we run around. It could relieve stress*,* and even the patient’s condition might improve.”* (P5, F, Clinic 4).

*“It could relieve us caregivers if there could be somewhere they spend the day. We wouldn’t always be stressed about their whereabouts as we would know they are safe out there. It would relieve us of having to monitor them and be unable to go anywhere closely.”* (P6, F, Clinic 1).

*“Also*,* there can be centres where we leave our patients if we go somewhere and collect them later when we come back.”* (P14, F, Clinic 4).

## Discussion

The results of this study support existing literature, indicating that caring for PLWMI is a challenging and stressful task. Family caregivers need comprehensive support, including assessing their well-being, environment, and resources. According to Phoen et al. [17], mental health assessment should be a priority for family caregivers and be provided with psychotherapeutic support when needed. In support of this, Foster et al. [37] stated that the assessment of family caregivers of PLWMI should be centred on continuous assessment practices involving identifying the caregivers’ presence in patient care and assessing the family needs. Moreover, the assessment should involve identifying the family’s strengths and weaknesses and the impact the mental illness has on the caregiver and the entire family.

Extant literature indicates home visits by mental healthcare professionals as one of the key needs for family caregivers of PLWMI [38]. Consistent with the literature, family caregivers in this study expressed the need to be checked at their homesteads, citing that it would allow the health workers to assess how the family caregivers cope and foster their therapeutic relationship as it conveys a sense of care and commitment. Mental health home visits offer safe, supportive care that benefits patients and their caregivers [39]. It is further explained that during home visits, nurses explore how to support family caregivers and their relatives diagnosed with mental illness while also enhancing their understanding of the family and the significance of their support. Furthermore, psychiatric community nursing helps improve family caregivers’ and patients’ quality of life by providing knowledge, skills, and practical support in their homes [40]. However, it is not cost-effective for a health worker to travel to a caregiver’s home for a consultation instead of seeing them at the clinic, where they can consult many individuals per day [41].

The findings of this study revealed that family caregivers need psychological support to deal with the psychological strain. They expressed that they are emotionally overwhelmed due to the strain of taking care of PLWMI, hence the need for counselling. According to Anokye [42], family caregivers of patients diagnosed with mental illness need counselling to achieve a stable and good mental state to execute the caregiving role competently. Moreover, the family caregivers have a dire need for positive feelings that their family members diagnosed with mental illness are recovering well. Olawande et al. [43]., in agreement with this study’s findings, revealed that most caregivers in their study expressed the need to be counselled, and the authors supported that seeking professional counselling from trained personnel is one of the key steps toward coping with caring for an individual diagnosed with mental illness. Literature indicates that family caregivers provided with stress management training experience increased levels of positive feelings [44].

The family caregivers pointed out that they do not have details about the mental illness their patient is diagnosed with and expressed their need to be provided with information to close the knowledge gap. Consistent with the study results, studies conducted by Moudatsou et al.. and Shamsaei et al. [45, 46]. indicated the caregivers’ need for information about the patient’s mental illness diagnosis, prognosis, and the management of the mental condition. Studies highlight that knowledge about mental illness is notably linked with the quality of life of caregivers of PLWMI Leng et al. and Moudatsou et al. [45, 47]. According to Moudatsou et al. [45]., inadequate knowledge about mental illness leads to increased family caregivers’ sense of burden and suggests that understanding the attributes of mental illness can assist the caregivers in recognizing their relative’s mental health issues and evaluating and accepting the situation. The provision of psychoeducation can serve to give information about the mental condition, how to manage it, give emotional support, and implement stress management and coping strategies [48]. Furthermore, psychoeducation enhances the caregivers’ skills to improve their caregiving roles, thereby increasing their caregiving capacity and decreasing the stressful burden of caregiving [44].

The need for grants and allowances for family caregivers of PLWMI emerged as one of the themes in this study’s findings. The caregivers expressed their wish for the government to assist them with money to cover their expenses which they said could reduce their burden emanating from financial constraints. There are no respite care services or inpatient rehabilitation centres in Botswana. So, family caregiving is informal, with the families being the mainstay of informal caregiving. The bearing of care (in all its aspects, e.g., financial, etc.) is incumbent upon the families of PLWMI; hence, the emerging theme from the interviews is that they need financial assistance. This is consistent with the findings of a study conducted by Shamsaei et al. [46]., which showed that family caregivers need financial support because of financial limitations, which were conveyed as the major challenge to most of the caregivers of PLWMI. Additionally, according to Amini et al. [49], financial support to family caregivers is a key factor in the sustainability of caregivers. Olawande et al. [43] revealed government financial support as one of the popular approaches in assisting family caregivers cope with overwhelming caregiving stress. Financial support lessens the family caregivers’ financial difficulties and attenuates their perceived burden, facilitating an improved quality of life [47].

According to the family caregivers’ experiences, they need support to access mental healthcare services. Some caregivers reported that they stay a considerable distance from health facilities, making it difficult to access mental health services because of a lack of transport and sometimes due to public transport operators refusing to assist them due to the patient’s behaviour. The findings of this study align with the results of a study conducted by Olawande et al. [43], which revealed that caregivers had to travel long distances to access mental health services, in addition to facing high costs for medication. The study also called for government and mental health professionals’ interventions through building more psychiatric hospitals and reinforcing national health insurance to increase access to mental health services. According to Ohtake et al. [39], increased access to family caregivers who struggle to travel long distances for mental health services can be achieved through home visits.

Consistent with the literature, the family caregivers noted the need for respite care to relieve family members’ burdens. They suggested that the government or town council should build centres to drop their patients off temporarily while they engage in other activities. They emphasized that it could relieve their stress and assist in the patient’s recovery. In agreement with our study’s results, caregivers in a study conducted by O’Neill et al. [50]. pointed out the need for respite care for their relatives diagnosed with mental illness as a coping strategy to deal with the caregiving burden. Additionally, findings in a study conducted by Harris et al. [51]. confirm the considerable service need for respite care among family caregivers of individuals diagnosed with mental disorders.

Research conducted in Lobatse reveals that caregivers view their circumstances as a divine test, frequently seeking solace through prayer [52]. Moreover, the socio-cultural landscape presents significant challenges, with a prevailing expectation that caregivers, particularly women -must do everything possible to support their family members, even when resources and support are limited. This immense pressure can have detrimental effects on their overall well-being. The findings of the current study can be used to revise the existing guidelines on disability support and to extend assistance to caregivers. These findings may also impact the upcoming Botswana Mental Health Policy of 2003 review.

### Limitations of the study

This is a small-scale study, which limits the generalizability of the results. However, the findings offer unique, in-depth insights into the family caregivers studied. The authors included participants who could only communicate in the country’s two official languages, which might have introduced bias. As well the study focused on general mental illness, creating a gap for further studies on specific psychiatric disorders. Using the purposive sampling technique of family caregivers of PLWMI risks getting biased results, especially if the selected sample does not represent the wider population under study. The participants’ selection bias was addressed by stating the inclusion and exclusion criteria. Qualitative research has the potential for participant bias, which can distort data authenticity and hinder the acquisition of genuine insights. The researcher prevented such bias by establishing rapport and presenting a nonjudgmental attitude that promoted participants’ openness. Researcher subjectivity is one of the potential limitations of the study. It was addressed by sharing the findings with the research supervisors for critical appraisal and allowing researchers to reflect on their assumptions and preconceptions that may impact the research. Additionally, the researcher maintained a reflective journal to document their thoughts and reflections while sharing it with research supervisors to enhance transparency and minimize subjectivity.

### Implications for nursing practice

The study suggests that family caregivers of PLWMI need early comprehensive assessment to identify their needs and interventions to support them. The aim of the assessment should be to enhance early detection and relief of psychological burden faced by the family caregivers, develop group or individually tailored approaches aligning with the identified family caregivers of PLWMI’s needs, and assist them in developing effective coping strategies. The family caregivers in the context of this study should be provided with educational, tangible, and psychosocial interventions to support them mitigate the caregiver burden they experience Additionally, they should be referred to appropriate support organizations to allow early enrolment in appropriate programs. The authors recommend developing guidelines to enhance the implementation of the recommended interventions.

## Conclusions

Overall, the findings of our study suggest that primary care of a family member diagnosed with mental illness at home is remarkably challenging and demanding, and has deleterious effects on the caregivers’ financial, social, and emotional well-being. The study unearthed the needs of family caregivers in caring for their family members living with mental illness which include the need for interventions to assist family caregivers, family caregivers’ need for informational support, and family caregivers’ need for tangible support. These findings indicate the necessity for guidelines to strengthen the well-being of family caregivers and provide them with adequate emotional, financial, and instrumental support.

## Electronic supplementary material

Below is the link to the electronic supplementary material.

Supplementary Material 1

## Data Availability

Data on interviews are available from the corresponding author upon reasonable request.

## Abbreviations

PLWMI: People living with mental illness
LDHMT: Lobatse District Health Management Team
MoH: Ministry of Health
NWU: HREC-North-West University Health Research Ethics Committee
