# Supplementary material for: Exploring the needs of family caregivers of people living with mental illness: a qualitative study in Lobatse, Botswana
Source: BMC Nurs. 2025 Feb 17;24:183. doi: 10.1186/s12912-025-02813-7 (PMC11834672; doi:10.1186/s12912-025-02813-7)
Supplement: Supplementary file 1 — Supplementary Material 1 [file 12912_2025_2813_MOESM1_ESM.docx]

**INTERVIEW GUIDE**

Questions

1. What are your needs in caring for a person living with a mental illness?
2. How do you cope with caring for this patient?
3. What can be done to assist you in coping with the strain of caring for a person living with mental illness?
